# Removal of Diclofenac and Heavy Metal Ions from Aqueous Media Using Composite Sorbents in Dynamic Conditions

**DOI:** 10.3390/nano14010033

**Published:** 2023-12-21

**Authors:** Daniela Fighir, Carmen Paduraru, Ramona Ciobanu, Florin Bucatariu, Oana Plavan, Andreea Gherghel, George Barjoveanu, Marcela Mihai, Carmen Teodosiu

**Affiliations:** 1 Department of Environmental Engineering and Management, “Gheorghe Asachi” Technical University of Iasi, 73 D. Mangeron Street, 700050 Iasi, Romania; daniela.fighir@academic.tuiasi.ro (D.F.); cpadur2005@yahoo.com (C.P.); ramona.ciobanu@student.tuiasi.ro (R.C.); fbucatariu@icmpp.ro (F.B.); oana.plavan@tuiasi.ro (O.P.); andreea.tereza@yahoo.com (A.G.); george.barjoveanu@academic.tuiasi.ro (G.B.); 2 “Petru Poni” Institute of Macromolecular Chemistry, 41A Grigore Ghica Voda Alley, 700487 Iasi, Romania

**Keywords:** fixed-bed column, adsorption, heavy metals, diclofenac, composite sorbents

## Abstract

Pharmaceuticals and heavy metals pose significant risks to human health and aquatic ecosystems, necessitating their removal from water and wastewater. A promising alternative for this purpose involves their removal by adsorption on composite sorbents prepared using a conventional layer-by-layer (LbL) method or an innovative coacervate direct deposition approach. In this study, four novel composite materials based on a silica core (IS) and a polyelectrolyte coacervate shell were used for the investigation of dynamic adsorption of three heavy metals (lead, nickel and cadmium) and an organic drug model (diclofenac sodium salt, DCF-Na). The four types of composite sorbents were tested for the first time in dynamic conditions (columns with continuous flow), and the column conditions were similar to those used in wastewater treatment plants. The influence of the polyanion nature (poly(acrylic acid) (PAA) vs. poly(sodium methacrylate) (PMAA)), maintaining a constant poly(ethyleneimine) (PEI), and the cross-linking degree (r = 0.1 and r = 1.0) of PEI chains on the immobilization of these pollutants (inorganic vs. organic) on the same type of composite was also studied. The experiments involved both single- and multi-component aqueous solutions. The kinetics of the dynamic adsorption process were examined using two non-linear models: the Thomas and Yoon–Nelson models. The tested sorbents demonstrated good adsorption capacities with affinities for the metal ions in the following order: Pb^2+^ > Cd^2+^ > Ni^2+^. An increase in the initial diclofenac sodium concentration led to an enhanced adsorption capacity of the IS/(PEI-PAA)_c-r1_ sorbent. The calculated sorption capacities were in good agreement with the adsorption capacity predicted by the Thomas and Yoon–Nelson models. The substantial affinity observed between DCF-Na and a column containing composite microparticles saturated with heavy metal ions was explained.

## 1. Introduction

There are economic and social concerns regarding water contamination with pharmaceutical compounds and heavy metal ions that originate from anthropogenic activities [1]. Persistency, toxicity, stability, low biodegradability and bio-accumulative behavior are some of the main characteristics associated with pharmaceuticals and heavy metal ions present in aquatic ecosystems [2,3]; these compounds are part of the priority/emerging pollutants and represent significant threats to human health, aquatic ecosystems and requirements for further water/wastewater treatment. Harmful heavy metal cations such as lead, nickel, cadmium and pharmaceuticals such as diclofenac (DCF) are often found in wastewater, thus representing the focus of numerous adsorption studies [4,5,6,7,8]. The removal of these micropollutants from wastewater to make it reusable is a mandatory requirement. In addition, the recovery of heavy metals from waste effluents provides a valuable resource for these indispensable materials that are widely used in all sectors of society and constitute key materials for renewable energy development [9,10].

To date, adsorption has been extensively used in wastewater treatment processes due to its well-known advantages, e.g., cost-effectiveness, high efficiency and ease of operation [11,12,13]. Another strong advantage of this process is the availability of a wide range of sorbents and the possibility of regeneration and reusability of the used sorbents. Several sorbent materials, including activated carbon [14,15], composites with activated carbon [16], zeolites [17,18], biochar [19], biosorbents [20,21], chitosan [22,23], clays [24], metal–organic frameworks [25,26], activated hydrochars [27], carbon nanotubes [28], graphenes [29,30], polymers [8,31] and nano-adsorbents [32,33,34], have been investigated for the removal of different organic/inorganic priority and emerging pollutants. Among them, composite sorbents may represent the best choice to deal with toxic pollutants such as pharmaceuticals and heavy metal ions. The composites successfully integrate different properties of two or more materials into one, for example, the combination of a large number of functional groups of organic materials with the hardness of inorganic materials [31]. For these reasons, the composite polyethyleneimine (PEI)/silica (SiO_2_) sorbents synthetized using different methods (layer-by-layer technique, coacervate deposition, grafting, dispersion, etc.) have gained popularity in wastewater treatment applications. Thus, Morosanu et al. [10] compared the sorption performances of twelve PEI/SiO_2_ composite sorbents prepared by a classic layer-by-layer (LbL) method and a novel one-pot deposition approach to remove cadmium ions from aqueous solutions in a batch mode. The sorbents obtained using the one-pot approach exhibited maximum sorption capacities, between 67 and 82.8 mg/g polymer from composite, superior to sorbents obtained by the LbL method. In another study, Bucatariu et al. [31] tested four PEI/SiO_2_ nanocomposites for their sorption properties of different heavy metal ions (Cu^2+^, Pb^2+^, Ni^2+^ and Fe^2+^). Sorption tests showed a good capacity for Pb^2+^ and Fe^2+^/Fe^3+^ removal for all types of composites. The same authors investigated two LbL strategies to construct a dynamic organic architecture on silica microparticles with ultra-fast and high loading/release capacity for copper ions. The sorption experiments recorded a maximum value of approximately 12 mg Cu^2+^/g composite [35]. Meanwhile, the nanoparticles of hyperbranched PEI/Si synthesized through a water-based sol–gel approach exhibited Langmuir sorption capacities for lead, copper and zinc of 833.3, 502.5 and 193.4 mg/g, respectively, in batch conditions [36].

As an alternative to batch sorption studies, column sorption studies ensure a comprehensive understanding of the dynamics of the sorption process for large-scale operations such as those in wastewater treatment plants. To evaluate the dynamic sorption performance of fixed-bed columns, it is necessary to analyze the breakthrough curves. However, few studies have been reported in the literature so far related to the removal of lead, nickel, cadmium and diclofenac in continuous-flow-mode operations. In one example, a column adsorption study of DCF, heavy metal ions (Cd^2+^, Ni^2+^) and oxyanions (arsenate and chromate ions) on an amino-functionalized lignin-based microsphere sorbent (A-LMS) was carried out. The sorption capacities achieved according to the breakthrough point were as follows: DCF (151.13 mg/g) >> Cd^2+^ (58.1 mg/g) > Cr^6+^ (54.1 mg/g) > As^5+^ (50.9 mg/g) >> Ni^2+^ (42.9 mg/g) [12]. Also, fixed-bed column experiments showed a high loading capacity for DCF from aqueous solutions using a hybrid material (aluminium–hexadecyltrimethyl ammonium bromide–sericite (AHS) and aluminium–alkyldimethylbenzyl ammonium chloride–sericite (AAS)). The loading capacities of DCF were found to be 0.561 and 1.056 mg/g for AAS and AHS, respectively [37]. In another study, a polyurethane foam cube (PUC) was employed to investigate the removal of Ni^2+^ from an aqueous solution. A fixed-bed sorption study was carried out by varying the sorbent bed height to 5 cm and 10 cm and using feed flow rates of 2 and 6 mL/min. The maximum Ni^2+^ adsorption by PUC was found to be 24.39 mg/g, at a flow rate of 2 mL/min and bed height of 10 cm [38]. Alternatively, Ghasemabadi et al. [39] studied the capacity of a mixture of magnetic graphene oxide and sand to remove Cr^6+^, Pb^2+^, As^3+^ and Cd^2+^ ions from aqueous media. Results of fixed-bed experiments showed that the proposed media was efficient for Pb^2+^ removal with a sorption capacity of 0.189 mmol/g, while Heidarzadeh-Samani et al. [40] obtained a Cd^2+^ removal efficiency of 82% in a fixed-bed column packed with starch-g-poly(acrylic acid)/cellulose nanofiber bio-nanocomposite hydrogel.

The main objectives of this study were to: (a) investigate the sorption capacities of four novel composite sorbents composed of a silica core (IS) and a polyelectrolyte coacervate shell in dynamic conditions; (b) assess the removal of three heavy metal ions (lead, nickel and cadmium) and an organic model pollutant, diclofenac sodium salt (DCF-Na), from single- and multi-component aqueous media under breakthrough conditions and variable DCF-Na concentrations; and (c) study the kinetics of the sorption processes in dynamic conditions by comparing experimental data against two non-linear models: the Thomas model and Yoon–Nelson model.

The novelty of this study consists of:

- Conducting experiments in dynamic conditions with four novel composite materials obtained through coacervate deposition. Experimental conditions were conceived to investigate the influence of specific process parameters (e.g., continuous column flow) on the sorption process effectiveness (sorption capacities at breakthrough point), in conditions similar to those used in wastewater treatment plants;
- Using multi-component aqueous solutions in sequential and simultaneous conditions. The multi-component solutions were made up of three heavy metal ions and an organic pollutant at concentrations that would simulate real wastewater conditions;
- Studying the influence of the polyanion nature on composite materials’ performance in immobilizing these pollutants. For this, several approaches were tested during the one-pot syntheses: (a) poly(acrylic acid) (PAA) and poly(sodium methacrylate) (PMAA) were compared as complexation agents for PEI while maintaining the poly(ethyleneimine) (PEI) concentration constant, and (b) using two PEI chain cross-linking degrees (r = 0.1 and r = 1.0).

## 2. Materials and Methods

### 2.1. Materials

All chemical reagents used in the study were of analytical-grade purity and were used as follows: heavy metal salts Cd(NO_3_)_2_·4H_2_O, NiSO_4_·6H_2_O and Pb(NO_3_)_2_ (Fluka, Buchs, Switzerland); diclofenac sodium salt (DCF-Na) (98%, Acros Organics, Geel, Belgium); NaOH and HNO_3_ (Merck, Darmstadt, Germany). Silica particles (Daisogel) with a diameter of 40–60 microns were purchased from Daiso Chemical Co. (Osaka, Japan). Poly(sodium methacrylate) (PMAA) (M_w_ = 1800 g/mol), polyacrylic acid (PAA) (M_w_ = 10,000 g/mol) and branched poly(ethyleneimine) (PEI) (M_w_ = 25,000 g/mol) were obtained from Sigma-Aldrich, Germany (Merck, Darmstadt, Germany).

### 2.2. Composite Materials’ Synthesis

The IS/(PEI-PAA)_c_ and IS/(PEI-PMAA)_c_ composite sorbents were synthesized following the methodology outlined in our previous research [10]. Specifically, 5.0 g of silica microparticles was suspended in 12 mL of 1 mol/L PEI, followed by the gradual addition of 6 mL of either PAA or PMAA, also at a concentration of 1 mol/L, under vigorous agitation until the solution achieved transparency. To achieve the desired cross-linking ratio (r), namely weak cross-linking (r = 1:10 = [−CHO]:[−NH_2_]) or strong cross-linking (r = 1:1 = [−CHO]:[−NH_2_] = 1:1) within the composite shell, a specific volume of glutaraldehyde (GA) (2.5% *w*/*v*) was determined based on the initial concentration of PEI. Subsequently, to facilitate the extraction of the non-cross-linked polyelectrolyte chains, the core–shell composite microparticles were subjected to treatment in a strong basic (1 mol/L NaOH) or acidic (1 mol/L HCl) medium. The syntheses protocols included an investigation of the influence of polyanion nature on the immobilization of different classes of pollutants (inorganic vs. organic) on the same type of composite. Namely, [poly(acrylic acid) (PAA) and poly(sodium methacrylate) (PMAA)] polyanions were tested as PEI complexation agents while maintaining a poly(ethyleneimine) (PEI) concentration constant. Another factor, the variation of the degree of cross-linking of PEI chains (r) was also investigated (r = 0.1 and r = 1.0). By using these conditions, PAA, a hydrophilic polyelectrolyte, and PMAA, a less hydrophilic polyelectrolyte, combined with weak (r = 0.1) and a strong (r = 1.0) PEI cross-linking degrees, generated four types of polymeric shells around the inorganic core. During testing, these core–shell inorganic–organic microparticles displayed different sorption capacities for each class of pollutants, as functions of the polyanion nature and degree of cross-linking. These materials were previously characterized using scanning electron microscopy, FTIR-ATR, thermogravimetric analysis and polyelectrolyte and potentiometric titrations [10]. The main characteristics of these four composite materials are presented in Table 1.

Although the synthesis, characterization and batch sorption tests of these composites are already published, the dynamic sorption capacities have not been evaluated until now.

### 2.3. Dynamic Sorption Tests

The dynamic sorption tests were performed in OMNIFIT chromatographic columns (900 PSI), where approximately 700 mg of composite material was uniformly distributed. The sorption tests were conducted using the four types of composite sorbents obtained through coacervate deposition (Table 1). Aqueous solutions of metal ions and diclofenac sodium were introduced into the column by using a peristaltic pump at a constant flow rate of 2 mL/min.

The experimental methodology employed for the investigation of dynamic retention of metal ions and DCF-Na included the following steps: initially, the column was loaded with a solution containing 41 mg/L ions. The eluate was collected in 50 mL containers and subsequently subjected to analysis. Following this, the column material was treated with 1 M HNO_3_ solution to facilitate the removal of the sorbed Pb^2+^ ions. The material was then washed with distilled water and activated with a 1 M NaOH solution. This procedure was applied to solutions containing Cd^2+^ ions at 22 mg/L, Ni^2+^ ions at 12 mg/L and DCF-Na at 100 mg/L (in this order). This sorption–desorption cycle was performed four times for each composite material

The multicomponent pollutant sorption was investigated in two consecutive processes in the column. Firstly, an echimolar aqueous mixture formed from the three heavy metal ions (C_Cd2+_ = C_Ni2+_ = C_Pb2+_ = 0.1 mM) was elluated in a column containing 700 mg sorbent. Then, the heavy-metal-ion-loaded column was used for the sorbtion of DCF-Na molecules. The influent concentration of this solution was kept constant throughout the experiment at 148 mg/L (or 0.5 mM). Finally, the fully exhausted sorbent for the three heavy metal ions and DCF-Na was treated with HNO_3_ 0.5% (*w*/*w*%) and NaOH (1 M) to remove all immobilized pollutants on the composite surface.

The eluates from the mono- or multi-tests were analyzed for their heavy metal concentrations using atomic absorption spectroscopy (contrAA 800, Analytik Jena, Jena, Germany). The DCF-Na concentration was analyzed via a direct spectrophotometric method at a wavelength of λ = 276 nm [41], using an Analytik Jena Specord 210 Plus spectrophotometer (Analytik-Jena, Bucharest, Romania). The calibration curves were validated by certified reference materials, namely DCF-Na 1 mg/mL in methanol from LGC and a multi-element metal standard solution from Merck.

The sorption capacity in dynamic conditions was calculated using the following formula [42]:

(1) $$q_{dyn}=\frac{C_{0}}{m}\int_{0}^{V_{i}}(1-\frac{C_{t}}{C_{0}})dV$$

where m is the mass of the sorbent bed (g), C_0_ is the initial concentration of the component under investigation (mg/L), C_t_ is the effluent concentration at the time of interest (mg/L) and V is the volume (L).

The sorption efficacy of the composite materials was evaluated by using the mass transfer zone (MTZ) concept, which denotes the length of the adsorption zone in the column and which is calculated according to Equation (2) [43]:

(2) $$MTZ=Z\times \frac{t_{s}-t_{b}}{t_{s}}$$

where Z represents the height of the composite material bed within the column, while t_b_ and t_s_ correspond to the breakthrough and saturation times, respectively.

### 2.4. Adsorption Data Modeling

The adsorption isotherms are very important for the design of a sorption system, as these models provide information about the sorption capacity and possible removal efficiencies. The kinetics of the dynamic sorption processes were assessed by fitting the experimental data to two different non-linear models: the Thomas and Yoon–Nelson models. The constants of the non-linearized forms were obtained using Origin 2019b software (OriginLab Inc., Northampton, MA, USA, 2019).

The Thomas model, a frequently used theoretical framework, serves as a fundamental tool for evaluating column performances and predicting the concentration–time profiles of complete breakthrough curves. This model postulates that sorption is primarily constrained by mass transfer dynamics at the interface, as opposed to chemical interactions among molecules. In our research, we used the Thomas model to determine the sorption capacities of the four composite materials within the column [44]. The model is mathematically represented in non-linear form by the following equation:

(3) $$\frac{C_{t}}{C_{0}}=\frac{1}{1+exp[\frac{K_{th\times q_{e}\times m}}{Q}-{K_{th}\times C}_{0}\times t]}$$

where C_0_ and C_t_ (mg/L) represent the initial concentration of the component in the influent and at time t (min), respectively; K_th_ (L/(min·mg)) is the Thomas constant; q_e_ (mg/g) is the maximum sorption capacity in the column; m (g) is the mass of the composite material; and t (min) is time.

The Yoon–Nelson model assumes that the adsorption probability decrease rate for each adsorbate molecule is directly proportional to adsorbate breakthrough probability and adsorbate sorption probability [45]. The analysis of breakthrough curves obtained using the Yoon–Nelson model involves determining the model parameters, k_YN_ and τ, based on the non-linear model as expressed by Equation (4):

(4) $$\frac{C_{t}}{C_{0}}=\frac{1}{1+e^{k_{YN}(\tau -t)}}$$

where C_0_ and C_t_ (mg/L) represent the initial concentration of the analyzed component in the influent and at time t (min), respectively; k_YN_ (1/min) is the Yoon–Nelson constant; and τ (min) is the time at which the concentration of metal ions and DCF-Na in the effluent is at 50% of the breakthrough concentration.

## 3. Results

### 3.1. Breakthrough Curves’ Analysis

**Figure 1:**
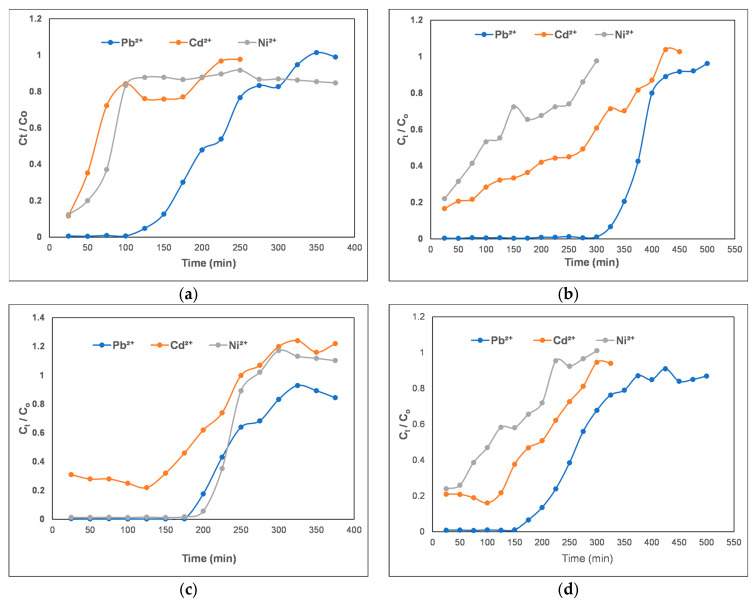
Breakthrough curves of the investigated composite materials: (**a**) IS/(PEI-PAA)_c-r0.1_; (**b**) IS/(PEI-PMAA)_c-r0.1_; (**c**) IS/(PEI-PAA)_c-r1_; (**d**) IS/(PEI-PMAA)_c-r1._

**Figure 2:**
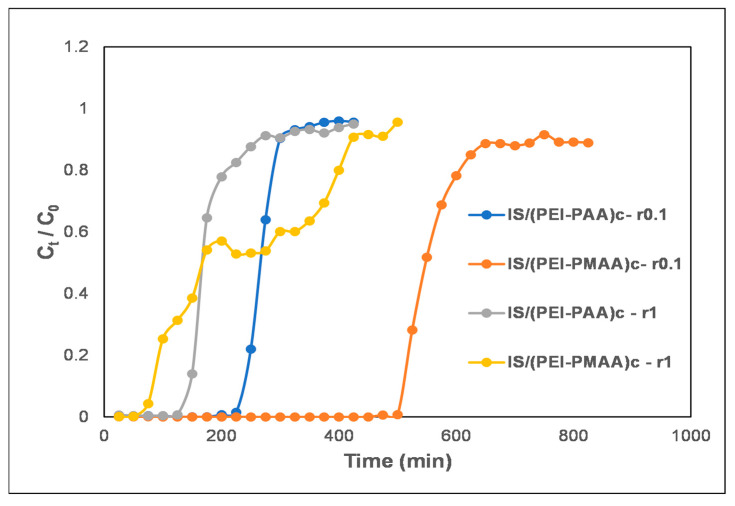
Breakthrough curves of the investigated composite material for DCF-Na sorption.

The experimental results, derived from the continuous flow of aqueous solutions containing metal ions and DCF-Na through a fixed-bed column, were employed to generate breakthrough curves by plotting the effluent concentration relative to the initial concentration against the column’s operational time. Figure 1 illustrates the breakthrough curves achieved in the retention of Pb^2+^, Cd^2+^ and Ni^2+^ metal ions, while Figure 2 presents the breakthrough curves for DCF-Na retention on the four composite materials, for the single-component pollutant aqueous solutions.

The assessment of fixed-bed sorption column performance relies on the analysis of breakthrough curves, which involve the calculation of key parameters [46,47] like the breakthrough time (t_b_) and breakthrough volume (V_b_), representing the moment when the effluent concentration reaches 10% of the initial concentration (C_t_/C_0_ = 0.1). Saturation time (t_s_) and saturation volume (V_s_) describe the time and volume at which the effluent concentration stabilizes at a constant value of 90% of the initial concentration (C_t_/C_0_ = 0.9). In cases where V_b_ is regarded as the initial point on the curve, the approximation is C_t_/C_0_ = 0.2. These parameters provide valuable insights into the efficiency of the sorption process within the column.

As observed in Figure 1, the breakthrough curves for Pb^2+^ ions on all four composite materials exhibit the characteristic “S” shape, resulting in more extended breakthrough times and higher sorption capacities as compared to the other heavy metal ions. These capacities fall within the range of 22.3 to 41.44 mg/g of composite, indicating an elevated affinity of the composite materials for Pb^2+^ ions. This trend is further substantiated by the shorter breakthrough times observed for the other two cations. Our data analysis supports the conclusion that the affinity for the metal ions follows the order Pb^2+^ > Cd^2+^ > Ni^2+^.

This affinity of heavy metals for the four novel sorbents is due to the fact that, following the basic treatment (regeneration), the amine groups in the material structure can form, through non-participating electrons, coordinative bonds with the metal ions. The number of adsorbed ions depends on the structure and degree of cross-linking of the composite material.

In terms of performance, IS/(PEI-PMAA)_c-r0.1_, despite having a lower cross-linking degree compared to IS/(PEI-PMAA)_c-r1_, demonstrates the higher sorption capacities for the heavy metal ions. This superior retention capacity can be attributed to its more porous structure, enabling heavy metal ions to interact with a greater number of functional groups.

In Table 2, it is evident that in all instances where t_b_ is exceptionally short, and breakthrough occurs within the initial 25 min, it results in an expansion of the MTZ towards the proximity of the sorbent bed’s height. This extension of MTZ leads to the elongation of the breakthrough curve, accompanied by a reduction in sorption capacity. Lower MTZ values indicate low mass transfer resistance and a correspondingly limited axial dispersion [43].

In Figure 2, the breakthrough curves for sodium diclofenac are presented for all four materials. It should be noted that DCF-Na was passed through the column during the fourth sorption–desorption cycle, while creating an acidic environment on each of the four studied materials. In general, from the data in Table 2, it can be observed that the adsorption of DCF-Na occurs at high breakthrough times, ranging between 81 and 508 min. Consequently, the sorption capacities are very high. Furthermore, on the IS/(PEI-PMAA)_c-r0.1_ material, a larger volume of solution was required for breakthrough, which occurred at a volume of 1016.81 mL and a time of 508 min. This phenomenon may be attributed to the structure of PMAA, which is a more hydrophobic polyanion compared to PAA, due to the presence of a methyl group in each structural unit of the PMAA polymeric chain.

### 3.2. Initial DCF-Na Concentration Influence

**Figure 3:**
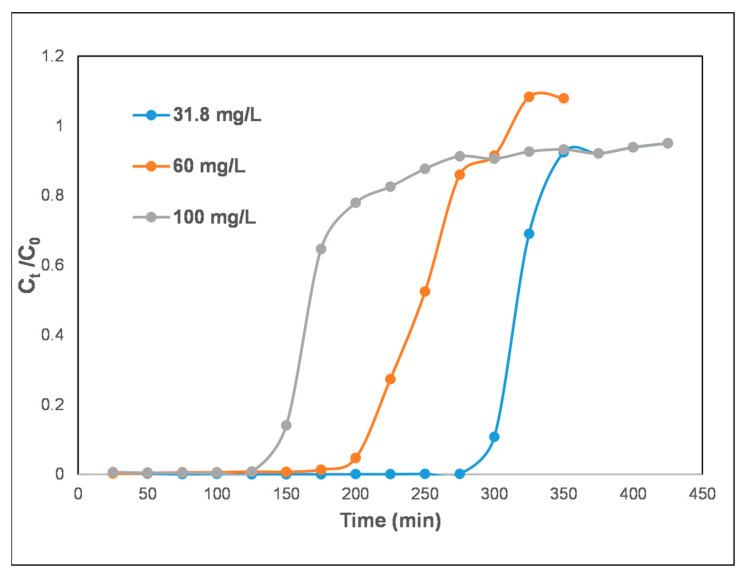
Breakthrough curves of DCF-Na sorption at varying initial concentrations.

The initial concentration’s influence was studied by performing tests on the IS/(PEI-PAA)_c-r1_ material at various initial DCF-Na concentrations (31.8 mg/L, 60 mg/L and 100 mg/L) while maintaining a constant bed height (40 mm) and a flow rate of 2 mL/min. The breakthrough curves presented in Figure 3, and the parameter values in Table 3, demonstrate that initial DCF-Na concentrations played a significant role in affecting the process’s performance.

As the initial concentration of DCF-Na increases, both the breakthrough volume and time decrease. This phenomenon is attributed to the faster occupation of active sites on the (PEI-PAA)_c-r1_ material by pollutant molecules. Furthermore, an increase in the initial concentration results in an enhanced maximum sorption capacity of the material. The increase in sorption capacity at higher concentrations may be due to a greater mass transfer force (potential), resulting from a larger concentration gradient between the liquid phase and the solid phase [48]. It can be observed that MTZ increases with the increasing initial concentration, from 0.78 to 1.86.

### 3.3. Column Kinetics Study

The Thomas and Yoon–Nelson models were used to non-linearly fit the experimental data obtained for the sorption of the heavy metal ions and then to calculate the specific kinetic parameters: K_th_ and q_max_ for the Thomas model, and k_YN_ and τ for the Yoon–Nelson model. These data are presented in Table 4 and Table 5, together with the correlation coefficients and reduced Chi-squared values, which are used as goodness-of-fit data.

The two models were initially tested and validated on the DCF-Na datasets for which multiple concentration data were available. For this, model parameters were obtained from non-linear data fit for two datasets (C_0_ = 60 and 100 mg/L) and then these parameters were used to predict data for the third concentration (31.8 mg/L). Then, predicted values were compared to the experimental data and the goodness of fit was evaluated through the Chi-squared and adjusted correlation coefficient (R^2^).

In case of the Thomas model, at first, the modeled values for q_max_ and K_th_ were used in linear regressions to predict the corresponding values at 31.8 mg/L, but the obtained values did not lead to a very good fit between the predicted and experimental data, which indicated that these kinetic parameters do not vary linearly with initial concentrations. Much better results were obtained for non-linear variations: a power function for q_max_ vs. initial concentration and an exponential decay fit for K_th_. These better q_max_ vs. C_0_ and K_th_ vs. C_0_ variations enabled a much better approximation (see values in Table 4). In the case of the Yoon–Nelson model, initial non-linear modeling for the 60 and 100 mg/L initial concentrations led to an almost constant value for k_YN_, which was then used to simulate C_t_/C_0_ values at a 31.8 mg/L initial concentration, which indeed indicated a very good fit.

The analysis of the presented data reveals that the adsorption capacity values closely approximate those derived from the analysis of breakthrough curves. This is demonstrated by the high R-squared (R^2^) correlation coefficient values, which vary from 0.786 to 0.996 and indicate a strong concordance between the experimental data and Thomas-generated data, affirming the model’s validity in describing the observed dynamic sorption behavior. Furthermore, varying the initial concentration of DCF-Na leads to a decrease in the values of the Thomas K_th_ constants and an increase in the values of sorption capacity.

Following the processing of experimental data in accordance with the Yoon–Nelson model, the data presented in Table 5 were obtained.

As can be seen in Table 5, the high values of the R^2^ coefficient and the low values of the χ^2^ factor led to the conclusion that the Yoon–Nelson model validates the data obtained through the processing of experimental data. Furthermore, when analyzing the different DCF-Na initial concentration values, it becomes apparent that with increasing initial concentration, the k_YN_ values and the values of time required for a 50% breakthrough (τ) decrease. This observation aligns with existing literature data [48]. The decrease in τ value can be attributed to the rapid saturation of active sites on the adsorbent at elevated concentrations, leading to a shorter residence time for the adsorption process [24].

### 3.4. Column Experiments for Multiple Pollutants’ Sorption onto the Same Silica/Polyelectrolyte Composites

**Figure 4:**
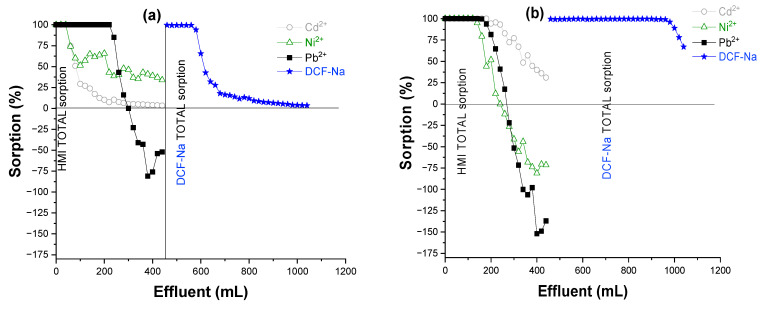

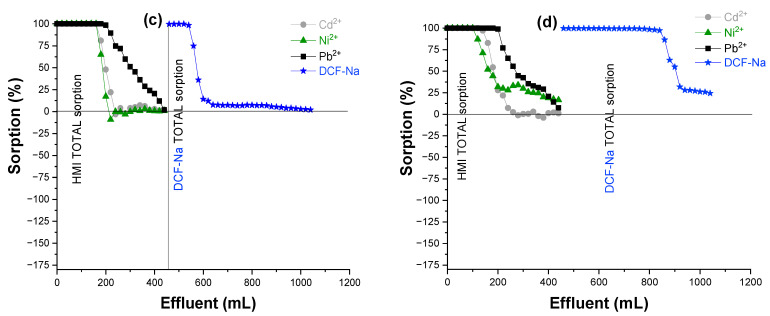
Sorption efficiency of Cd^2+^, Ni^2+^, Pb^2+^ and DCF-Na onto IS/(PEI-PAA)_c-r0.1_ (**a**), IS/(PEI-PMAA)_c-r1_ (**b**), IS/(PEI-PAA)_c-r0.1_ (**c**), and IS/(PEI-PMAA)_c-r1_ (**d**) from equimolar multi- (C_Cd2+_ = C_Ni2+_ = C_Pb2+_ = 0.1 mM) and mono-component (C_DCF-Na_ = 148 mg/L) influent solution carried out in two separated runs, where DCF-Na was retained by the exhausted composite in heavy metal ions.

To test the accessibility of all pollutant ions or molecules to the active sites of silica/polyelectrolyte core/shell particles, an equimolar aqueous mixture of three heavy metal ions (Cd^2+^, Ni^2+^ and Pb^2+^) and a DCF-Na aqueous solution were introduced successively into a column filled with different types of composites (Figure 4).

The dynamic sorption experiments showed that all tested heavy metal ions were immobilized on composite sorbents in non-competitive conditions (first 40–160 mL aqueous solution). In this case, the total sorption capacity is related to the polyelectrolyte materials used and cross-linking degree. The weakly cross-linked composite particles presented negative values for sorption capacities, as in the case of Pb^2+^ and Ni^2+^, which can be associated with a possible competition with Cd^2+^ ions (Figure 4a,b). This fact could be attributed to a higher concentration of the amino groups as compared with carboxyl groups (because non-cross-linked polyanion chains are being removed easily from the composite at low cross-linking degrees during synthesis).

In Figure 4, it can be observed that Pb^2+^ ions presented the highest sorbed amount (5.3–6.5 mg/g composite) as compared to the other two heavy metal ions for all composite types, this fact being attributed to the higher atomic mass of this element (206 g/mol) compared with Cd^2+^ (112 g/mol) and Ni^2+^ (59 g/mol). After the column was fully loaded with the three heavy metal ions (or exhausted), each type of composite sorbent was treated, in the same column, with a 148 mg/L DCF-Na aqueous solution, to further test the sorption capacity towards the other type of pollutant (organic). Heavy metal ions interacted with any of the functional groups in the polyelectrolytes that make-up the composite shell by forming coordinative bonds, while DCF-Na molecules, which contain ionized carboxylic groups, interacted with PEI amino groups by means of H-bonds and attractive electrostatic interactions. Moreover, DCF-Na molecules could interact by ionic exchange with already-sorbed heavy metal ions on the composite by replacing nitrates in the coordination sphere of the already-formed metallic complex. Also, hydrophobicity could act as a secondary force to retain DCF-Na inside the composite shell. This fact is demonstrated by the higher DCF-Na sorbed amount in the case of composites that contain PMAA in the organic shell, with PMAA being more hydrophobic than PAA due to the presence of methyl groups on each monomeric unit of the polymeric chain (Figure 4b,d). In Figure 4, we can observe that the weak and strong cross-linked IS/(PEI/PMAA) composite particles could retain a much higher amount of DCF-Na (84.57–109.94 mg/g composite) as compared with composites with PAA in the shell (21.14–25.37 mg/g composite). Also, the cross-linking degree has a significant influence on large organic molecules’ sorption, compared with small heavy metal ions, as the strongly cross-linked composites retain a small amount of DCF-Na. This fact demonstrates that DCF-Na sorption could be controlled by diffusion processes inside the composite shell. The dynamic sorption of each inorganic (Cd^2+^, Ni^2+^ and Pb^2+^) and organic (DCF-Na) pollutant, presented in Figure 4, demonstrates two major aspects: (i) all pollutants were totally retained inside the composite shell in non-competitive conditions, and (ii) there was no competition between heavy metal ions and organic molecules for active sorption sites inside the composite particles, due to the different sorption mechanisms (coordinative bonds in the case of heavy metal ions and electrostic/H-bond/hydrobics in the case of charged organic molecules) (Table 6).

For the complete removal (95–100% desorption) of all pollutants retained inside the column, the composite material was treated with 0.5% HNO_3_ and 4% NaOH. Thus, a new cycle of sorption/desorption of other pollutants could start, but only after the composite sorbents’ activation with 1 M NaOH in the case of heavy metal ion retention or 1 M HCl for negatively charged organic molecules.

### 3.5. Adsorption Mechanism

**Scheme 1:**
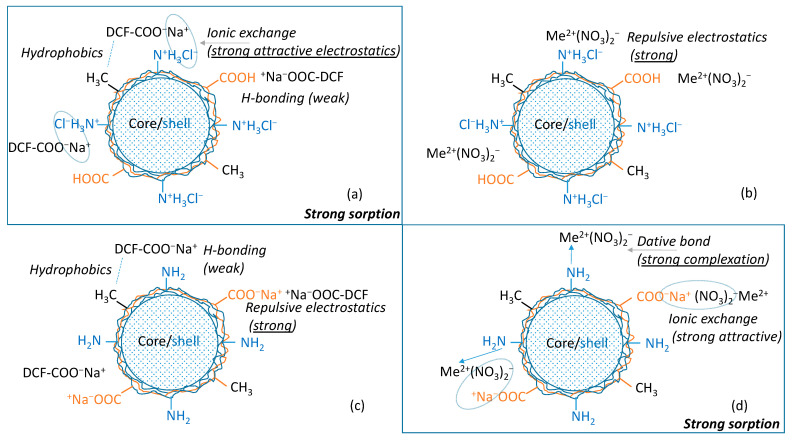
Principal types of interactions between DCF-Na (**a**,**c**) and heavy metal ions (**b**,**d**) with core/shell composite surface activated with HCl 0.1 M (**a**,**b**) and NaOH 0.1 M (**c**,**d**).

The pollutant (DCF-Na or Me^2+^) sorption process onto these core/shell sorbents is driven mainly by attractive electrostatic interactions, H-bond hydrophobics in the case of organic molecules (DCF-Na), and coordinative bonds and ionic exchange in the case of heavy metal ions (Me^2+^), as depicted in Scheme 1. DCF-Na molecules could interact by ionic exchange with already-sorbed heavy metal ions on composites by replacing nitrates in the coordination sphere of the already-formed metallic complex. Also, hydrophobicity could act as a secondary force to retain DCF-Na inside the composite shell. This fact is demonstrated by the higher amount of sorbed DCF-Na in the case of PMAA composites (compared to the PAA-containing ones), as PMAA is more hydrophobic than PAA due to the presence of methyl groups on each monomeric unit of the polymeric chain. Each efficient sorption process requires an optimum balance between all types of these interactions; therefore, all superficial functional groups (amino and carboxyl) must be activated prior to each immobilization process. This activation could be achieved with HCl aqueous solution for anionic charged organic molecules and with NaOH in the case of heavy metal ions (Scheme 1a,d). The ammonium salt could interact electrostatically with anionic species, while amino groups could be involved in dative bond formation due to the free “p” electrons of the nitrogen atom.

## 4. Conclusions

This study focused on the dynamic sorption processes involving three heavy metal ions (Pb^2+^, Cd^2+^, Ni^2+^) and one organic pollutant (DCF-Na) on fixed-bed sorbents consisting of four innovative core/shell composite materials (silica core and polyelectrolyte coacervate polymeric shell).

Experiments demonstrated that the composite sorbents had good sorption capacities for all tested pollutants, with the affinity for the metal ions following the order Pb^2+^ > Cd^2+^ > Ni^2+^. In terms of performance, IS/(PEI-PMAA)_c-r0.1_, despite featuring a lower degree of cross-linking in comparison to IS/(PEI-PMAA)_c-r1_, exhibited the highest sorption capacities toward all types of pollutants. Heavy metal ions interacted with any functional groups available on the polyelectrolyte shell by forming coordinative bonds, while DCF-Na molecules, which contain an ionized carboxylic group, interacted predominantly with PEI amino groups by means of H-bond and attractive electrostatic interactions.

The sorption capacity of the IS/(PEI-PAA)_c-r1_ material was examined under conditions of varying initial concentration. An increase in the initial pollutant concentration led to an improved maximum sorption capacity for the tested sorbent. This superior retention capacity may be attributed to its more porous structure, enabling heavy metal ions to interact with a greater number of functional groups.

The kinetics analysis revealed that the obtained sorption capacity values closely matched those derived from the breakthrough curves. The R-squared (R^2^) correlation coefficients ranged from 0.78 to 0.996, indicating a strong concordance between the experimental data and the Thomas and Yoon–Nelson non-linear models.

All pollutants were totally retained inside the composite shell in non-competitive conditions. There was no competition between heavy metal ions and organic molecules for active sorption sites inside the composite particles, due to the different sorption mechanisms.

The strong interaction of DCF-Na with a column loaded with heavy-metal-saturated composite microparticles was attributed mainly to a synergic effect based on: (1) the replacement of nitrate ion in the previously formed complex (PEI_4_→Me^2+^)/(NO_3_^−^)_2_ with DCF-Na carboxyl groups, and (2) hydrophobic interactions of DCF-Na with polyelectrolyte chains, especially PMAA.

## Figures and Tables

**Table 1:** Composite material characteristics.

Composite Material	q_PEI_ (mg/g Composite)	[CHO]:[NH_2_] (r) (Cross-Linking Degree)	C_exchange_ (meq/g Composite)
IS/(PEI-PAA)_c-r0.1_	103.2	1:10	2.40
IS/(PEI-PMAA)_c-r0.1_	103.2	1:10	2.40
IS/(PEI-PAA)_c-r1_	103.2	1:1	2.40
IS/(PEI-PMAA)_c-r1_	103.2	1:1	2.40

Note: IS-silica microparticles (Daisogel) with 40–60 μm average particle diameter; PEI = branched poly(ethyleneimine), M_w_ = 25,000 g/mol; PAA = poly(acrylic acid), M_w_ = 30,000 g/mol; PMAA = poly(sodium methacrylate), M_w_ = 9500 g/mol; q_PEI_—the amount of PEI deposited; (PEI-PAA)_c_ and (PEI-PMAA)_c_ = coacervate deposited directly on Daisogel via precipitation of the PEI-PAA or PEI-PMAA complex; C_exchange_ = exchange capacity, c—coacervate; r—ratio of cross-linking (molar ratio between amino groups (PEI) and aldehyde groups (GA) inside the cross-linked shell of composite).

**Table 2:** Parameters of the breakthrough curves.

Composite Material	Pollutant	V_b_, mL	t_b_, min	V_s_, mL	t_s_, min	q_dyn_ mg/g	MTZ
IS/(PEI-PAA)_c-r0.1_	Pb^2+^	282.9	141.5	630.1	315.05	22.33	2.20
	Cd^2+^	50	25	412.71	206.35	5.04	3.51
	Ni^2+^	50	25	458	229	3.466	3.11
	DCF-Na	470.73	235.36	597.61	298.80	88.319	0.74
IS/(PEI-PMAA)_c-r0.1_	Pb^2+^	661.9	331	867.1	433.6	41.44	0.95
	Cd^2+^	50	25	808.66	404.33	17.352	3.28
	Ni^2+^	50	25	556.8	278.4	4.23	3.64
	DCF-Na	1016.81	508.41	1471.40	735.70	230.90	0.93
IS/(PEI-PAA)_c-r1_	Pb^2+^	378	189	625	312.5	25.73	1.58
	Cd^2+^	50	25	481.20	240.60	8.09	3.58
	Ni^2+^	407.2	204	458.9	229	6.70	0.45
	DCF-Na	284.94	142.47	532.69	266.34	44.41	1.86
IS/(PEI-PMAA)_c-r1_	Pb^2+^	374.5	187.2	841,5	420.7	29.98	2.22
	Cd^2+^	50	25	582.70	291	10.83	3.66
	Ni^2+^	50	25	438.35	219.2	3.85	3.54
	DCF-Na	163.47	81.74	843.25	421.63	72.09	3.22

Note: V_b_, mL—breakthrough volume; t_b_, min—breakthrough time; V_s_, mL—saturation volume; t_s_, min—saturation time; q_dyn_ mg/g—adsorption capacity in dynamic conditions; MTZ—mass transfer zone.

**Table 3:** Parameters of the breakthrough curves for DCF-Na.

Composite Material	DCF-Na mg/L	V_b_, mL	t_b_, min	V_s_, mL	t_s_, min	q_dyn_ mg/g	MTZ
IS/(PEI-PAA)_c-r1_	31.8	559.7	279.84	694.89	347.44	26.52	0.78
	60	411.78	205.89	600.67	300.33	41.17	1.25
	100	284.93	142.46	532.68	266.34	44.41	1.86

Note: V_b_, mL—breakthrough volume; t_b_, min—breakthrough time; V_s_, mL—saturation volume; t_s_, min—saturation time; q_dyn_ mg/g—adsorption capacity in dynamic conditions; MTZ—mass transfer zone.

**Table 4:** Values of the Thomas model parameters for the studied dynamic model.

Composite Material	Pollutants	K_th_ L/mg·min	q_e_ mg/g	χ^2^	R^2^
IS/(PEI-PAA)_c-r0.1_	Pb^2+^	6.436 × 10^−4^	25.116	1.65 × 10^−3^	0.990
	Cd^2+^	1.060 × 10^−3^	4.181	1.60 × 10^−2^	0.786
	Ni^2+^	3.660 × 10^−3^	2.725	1.31 × 10^−2^	0.820
	DCF-Na	7.134 × 10^−4^	76.487	8.24 × 10^−4^	0.996
IS/(PEI-PMAA)_c-r0.1_	Pb^2+^	1.210 × 10^−3^	44.740	4.96 × 10^−4^	0.995
	Cd^2+^	4.269 × 10^−4^	14.497	5.79 × 10^−3^	0.925
	Ni^2+^	9.158 × 10^−4^	3.746	4.22 × 10^−3^	0.913
	DCF-Na	3.228 × 10^−4^	159.203	3.58 × 10^−3^	0.978
IS/(PEI-PAA)_c-r1_	Pb^2+^	7.634 × 10^−4^	28.686	4.15 × 10^−3^	0.973
	Cd^2+^	1.020 × 10^−3^	10.236	3.31 × 10^−2^	0.807
	Ni^2+^	9.010 × 10^−3^	7.900	5.69 × 10^−3^	0.979
	DCF-Na (31.8 mg/L)	3.410 × 10^−3^	29.088	9.23 × 10^−4^	0.994
	DCF-Na (60 mg/L)	9.384 × 10^−4^	42.169	1.74 × 10^−3^	0.991
	DCF-Na (100 mg/L)	5.244 × 10^−4^	49.599	6.02 × 10^−3^	0.967
IS/(PEI-PMAA)_c-r1_	Pb^2+^	5.086 × 10^−4^	32.549	2.75 × 10^−3^	0.980
	Cd^2+^	6.916 × 10^−4^	11.583	4.42 × 10^−3^	0.949
	Ni^2+^	1.290 × 10^−3^	3.827	3.47 × 10^−3^	0.954
	DCF-Na	9.015 × 10^−5^	67.289	9.04 × 10^−3^	0.898

Note: K_th_ (L/(min·mg))—the Thomas constant; q_e_ (mg/g)—the maximum sorption capacity in the column; χ^2^—chi-square statistic factor; R^2^—R-squared coefficient.

**Table 5:** Values of the Yoon–Nelson model parameters for the studied dynamic model.

Composite Material	Pollutants	Yoon–Nelson Model			
		k_YN_ 1/min	τ, min	χ^2^	R^2^
IS/(PEI-PAA)_c-r0.1_	Pb^2+^	2.665 × 10^−2^	212.335	1.65 × 10^−3^	0.990
	Cd^2+^	2.33 × 10^−3^	66.520	1.60 × 10^−2^	0.786
	Ni^2+^	4.387 × 10^−2^	79.483	1.31 × 10^−2^	0.820
	DCF-Na	7.135 × 10^−2^	267.703	8.24 × 10^−4^	0.996
IS/(PEI-PMAA)_c-r0.1_	Pb^2+^	5.030 × 10^−2^	378.238	4.96 × 10^−4^	0.995
	Cd^2+^	9.390 × 10^−3^	230.641	5.79 × 10^−3^	0.925
	Ni^2+^	1.099 × 10^−2^	109.256	4.22 × 10^−3^	0.913
	DCF-Na	3.229 × 10^−2^	557.204	3.58 × 10^−3^	0.978
IS/(PEI-PAA)_c-r1_	Pb^2+^	3.161 × 10^−2^	242.515	4.15 × 10^−3^	0.973
	Cd^2+^	2.240 × 10^−2^	162.849	3.31 × 10^−2^	0.807
	Ni^2+^	1.081 × 10^−1^	230.412	5.69 × 10^−3^	0.979
	DCF-Na (31.8 mg/L)	1.083 × 10^−1^	318.23	9.75 × 10^−4^	0.994
	DCF-Na (60 mg/L)	5.623 × 10^−2^	245.98	1.74 × 10^−3^	0.99
	DCF-Na (100 mg/L)	5.245 × 10^−2^	173.597	6.02 × 10^−3^	0.967
IS/(PEI-PMAA)_c-r1_	Pb^2+^	2.106 × 10^−2^	275.170	2.75 × 10^−3^	0.980
	Cd^2+^	1.522 × 10^−2^	184.271	4.42 × 10^−3^	0.949
	Ni^2+^	1.543 × 10^−2^	111.622	3.47 × 10^−3^	0.954
	DCF-Na	9.020 × 10^−3^	235.515	9.04 × 10^−3^	0.898

Note: k_YN_ (1/min)—the Yoon–Nelson constant; τ (min)—the time at which the concentration of metal ions and DCF-Na in the effluent is 50% of the breakthrough concentration; χ^2^—chi-square statistic factor; R^2^—R-squared coefficient.

**Table 6:** Total and individual amounts of pollutant immobilized on each composite in dynamic conditions (non-competitive conditions).

Sorbent	q_Cd2+_ (mg/g)	q_Ni2+_ (mg/g)	q_Pb2+_ (mg/g)	q_DCF_ (mg/g)	q_total_ (mg/g)
IS/(PEI/PAA)_c-r0.1_	0.640	0.337	6.505	25.372	32.854
IS/(PEI/PMAA)_c-r0.1_	3.840	1.180	5.322	109.940	120.282
IS/(PEI/PAA)_c-r1_	2.560	1.348	5.914	21.142	30.964
IS/(PEI/PMAA)_c-r1_	2.241	0.842	5.914	84.57	93.567

## Data Availability

The data presented in this study are available on request from the first author.
